# Late gestational exposure to dexamethasone and fetal programming of abnormal behavior in Wistar Kyoto rats

**DOI:** 10.1002/brb3.2049

**Published:** 2021-02-02

**Authors:** Christine Lalonde, Julie Grandbois, Sandhya Khurana, Alyssa Murray, Sujeenthar Tharmalingam, T. C. Tai

**Affiliations:** ^1^ Biomolecular Sciences Laurentian University Sudbury ON Canada; ^2^ Division of Medical Sciences Northern Ontario School of Medicine Sudbury ON Canada; ^3^ Department of Biology Laurentian University Sudbury ON Canada; ^4^ Department of Chem/Biochem Laurentian University Sudbury ON Canada

**Keywords:** depression, fetal programming, glucocorticoids, prefrontal cortex

## Abstract

**Introduction:**

Fetal programming was characterized a few decades ago, explaining the correlation of physiological phenotypes of offspring exposed to early‐life stress. High acute or chronic prenatal stress can overwhelm the enzymatic placental barrier, inducing transcriptional changes in the fetus that can result in different adverse behavioral and physiological phenotypes. The current study investigates the impact of exposure to the synthetic glucocorticoid, dexamethasone, during late gestation on behavioral outcomes.

**Methods:**

Pregnant Wistar Kyoto rats were given daily subcutaneous injections from gestational days 15–21 of either dexamethasone (0.9% NaCl, 4% EtOH, 100 µg kg^−1^ day^−1^) or were physically manipulated as naïve controls. Pups were raised normally until 17 weeks of age and underwent the Porsolt swim task and elevated plus maze for depressive and anxiety‐like behaviors, respectively. Neural tissue was preserved for genetic analysis using quantitative real‐time polymerase chain reaction.

**Results:**

Statistical analyses show significant disruption of behavior and genetic profiles of offspring exposed to dexamethasone in‐utero. Exposed animals spent more time immobile on the swim task and entered open arms of the elevated plus maze more often than their naïve counterparts. In the prefrontal cortex, there was a sex by treatment interaction on gene expression relevant to neural transmission in ryanodine receptor 2, as well as increased gene expression in SNAP25, COMT, and LSAMP in males prenatally exposed to dexamethasone compared with controls. Both dysregulated genes and behavior are linked to decreased anxiety and fear inhibition.

**Conclusion:**

Our results indicate adult offspring exposed to dexamethasone in‐utero have a tendency toward passive stress‐coping strategies and an inhibition of anxiety on behavioral tasks. Methyltransferase activity, synaptic activity, and cellular processes were disrupted in the prefrontal cortices of these animals. Specifically, genes involved in emotional response pathways were overexpressed, supporting the link between the behavioral and genetic profiles. Combined, we determine that dexamethasone offspring have adaptive predispositions when faced with novel situations, with increased immobility in the swim task and increased exploration on the elevated plus maze.

## Graphical abstract

This study examined the impact of prenatal exposure to the synthetic glucocorticoids on postnatal adult behavior. Behavioral tasks used to examine the anxiety and stress‐coping strategies. (A) Elevated plus maze. (B) Porsolt swim task.

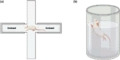

## 1 INTRODUCTION

In 1992, Barker and Hales introduced the research community to the concept that early‐life environmental conditions can influence the physiological phenotype of the offspring (Hales & Barker, 1992). They coined their early work, the ‘thrifty phenotype hypothesis,’ focusing on metabolism and diet. Their initial research showed that fetuses raised in a nutrient‐deficient environment would be predisposed to type 2 diabetes (Godfrey & Barker, 2001; Hales & Barker, 1992). Multiple studies over the past decades have supported this hypothesis, implicating insufficient nutritional intake as a driver for adult onset obesity, type 2 diabetes, hypertension, and overall metabolic syndrome markers (Lalonde, 2018; Marciniak et al., 2017; Tai & Tai, 2006). An evolutionary theory proposes that the fetal environment predicts conditions outside, providing an adaptive mechanism whereby offspring will increase fat stores, but this mechanism is maladaptive in a nutritionally dense environment (Del Giudice, 2012; Lalonde, 2018). Expansion on the thrifty phenotype hypothesis has provided research in other stressful uterine conditions, such as hypoxia, illness, and the introduction of exogenous glucocorticoid levels (Fajersztajn & Veras, 2017; Kapoor et al., 2008; Khurana et al., 2019; Thompson & Al‐Hasan, 2012). The impact of various maternal stress conditions that influence fetal development continues to support the theory behind the development of metabolic phenotypes, as well as other adverse diseases and behavior.

In response to a stressful environment, the mother will endogenously produce increased levels of stress hormones, glucocorticoids. The placenta has an enzymatic barrier to reduce a high concentration of glucocorticoids from gaining access to the fetus. The enzyme 11‐β hydroxysteroid dehydrogenase 1, or 11β1HSD, bidirectionally converts cortisol and corticosterone into inactive cortisone and 11‐dehydrocorticosterone, while 11β2HSD is unidirectional in inactivation and prevents about 80% of glucocorticoids from interacting with the fetus (Wyrwoll et al., 2015). 11β2HSD knockout animal models demonstrate adverse behavior related to depression and cognition, implicating glucocorticoid (GC) action on the nervous system (Wyrwoll et al., 2015). At high concentrations, GC can overwhelm the enzymatic barrier and bind to glucocorticoid receptors (GR) in the fetus, which are expressed in the cytoplasm of most cells, especially within different regions of the brain. The GC/GR complex then translocates to the nucleus and binds to glucocorticoid response elements (GRE) on various genes, initiating transcriptional activation and allowing for changes in the methylation status of CpG islands within GRE promoter regions (Clayton et al., 2019; Khurana et al., 2019; Provençal et al., 2019; Thomassin et al., 2001; Yang et al., 2012; Zannas & Chrousos, 2017). Hypothesized mechanisms for the change in local methylation status near GRE sites include a decrease in methyltransferases and an increase in ten‐eleven translocation methylcytosine dioxygenase (TET) in conjunction with the influx of GC (Zannas & Chrousos, 2017). Methylation can then activate or silence genes depending on the number and location of methyl groups (Moore et al., 2012; Werner, Gancarz, & Dietz, 2019). Synthetic glucocorticoids (sGC), such as betamethasone and dexamethasone, are often administered to pregnant mothers who are at risk for preterm birth (Grgić et al., 2003). The sGC mature the fetal lungs, increasing survivability of prematurely born offspring. Synthetic GC bypass the placental enzymatic barrier due to low binding efficiencies and bind to GR in high concentrations within the fetus. This process has been linked to fetal programming of metabolic disorder and changes in normative behavioral response to stressful situations in adult offspring (McGowan & Matthews, 2018; Moisiadis & Matthews, 2014b; Seckl, 2006).

Much research into methylation and fetal programming has focused on the hypothalamic–pituitary–adrenal (HPA) axis and the hippocampus (Moisiadis & Matthews, 2014a; Turner et al., 2010; Weaver et al., 2004). GR density, binding rate studies, and adrenalectomies have made clear associations in neurodevelopmental deficiencies (Cottrell & Seckl, 2009; Kapoor et al., 2006, 2008; Matthews et al., 2004). The HPA axis is involved in GC feedback and the hippocampus has high concentrations of GR, making both relevant targets of scientific research. When considering other aspects of mental health, however, other neural regions also play key roles and may be affected by GC concentrations.

The prefrontal cortex (PFC) has been implicated in a number of adverse behaviors and neural diseases. Early research involving neural imaging techniques that investigated structural changes, such as regional volume, and functional changes in activity patterns show a correlation between abnormalities in the PFC and clinical depression (George et al., 1994). Abnormal PFC activity is pronounced in individuals with depression and schizophrenia, and further research has linked the PFC to emotional control under stress, where abnormalities within the PFC are correlated to depressive‐like symptoms (Barch et al., 2003; Drevets, 2000; Lemogne et al., 2012). Treatment of depression is effective when the PFC is stimulated with transcranial magnetic stimulation, supporting the link between the PFC and depression (George et al., 2000). Additionally, the PFC is also known to be sensitive to high concentrations of GC and increased levels of stress, where exposure leads to impaired working memory and a reduction in gray matter (Arnsten, 2009). In consideration of the literature, we hypothesized that prenatal exposure to sGC would lead to the fetal programming of abnormal mental health inclinations and PFC dysregulation. Specifically, we hypothesize changes to coping mechanisms and anxiety‐like behaviors when faced with inescapable stress (Cartier et al., 2016; Cerqueira et al., 2005; Hiroi et al., 2016; McGowan & Matthews, 2018; Pascual et al., 2015).

## 2 MATERIALS AND METHODS

### 2.1 Ethics

All experimental protocols were approved by the Animal Care Committee of Laurentian University (AUP: 6013917) and were in accordance with the Canadian Council on Animal Care guidelines.

### 2.2 Animals and housing

Eight‐week‐old Wistar Kyoto rats (Charles River Laboratories) were housed in either pairs or triplets in Innocage® IVC disposable cages (Innovive Inc.). Cages contained cob bedding (Harklan) and enrichment tubes (Bio‐serv) and were placed into a HEPA filter Innorack® Rat airflow system (Innovive Inc.). Food (Teklad 22/5 Rodent Diet, Harklan) and water were available ad libitum. Animals were place on a 12:12 light‐dark cycle, with the light cycle starting at 6:00 a.m. Room temperature was maintained at 25°C and humidity at 53%.

### 2.3 Breeding

Male rats were introduced to three females for a period of five days. Females were checked daily for the presence of a vaginal plug and were then singly housed and weighed daily for the duration of their pregnancies. A total of four dams were utilized for DEX exposure, and a single dam was utilized to created naïve offspring and another for sham offspring.

### 2.4 Glucocorticoid treatment

On gestational day fifteen and onwards, pregnant dams were placed in one of two treatment conditions: daily subcutaneous injections of DEX (0.9% sodium chloride, 4% ethanol, and 100 µg/kg dexamethasone), sham injection (vehicle only), or naïve controls (physical manipulation only).

### 2.5 Offspring

Pups were weaned at three weeks of age, sexed, and housed in pairs or triplicates. Pups were raised under normal conditions until seventeen weeks of age, whereby they underwent behavioral testing.

### 2.6 Behavioral tasks

**FIGURE 1:**
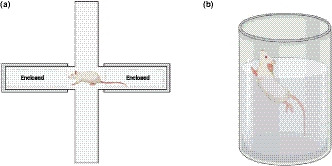
Behavioral tasks used to examine the anxiety and stress‐coping strategies. (a) Elevated plus maze. (b) Porsolt swim task. Figure created with biorender.com

All behavior was recorded utilizing video cameras and stored for scoring at a later time. Experimenters and observers were blind to treatment groups. Experimenters remained behind a black curtain to prevent distraction of the animals. All tasks were conducted during the light period, between 9:00 a.m. and 4:00 p.m. Animals underwent testing for a total of three days; the first task presented was the elevated plus maze (EPM) and then the Porsolt swim task (PST) on the following two days (Figure 1a,b).

#### 2.6.1 Elevated plus maze

The EPM task is designed to measure general anxiety associated with thigmotaxic behavior and exploration (Walf & Frye, 2007). Animals were individually transported into the testing room in a normal plexiglass cage with cob bedding. Each animal was placed in the center of the platform, 10.4 cm × 10.1 cm, facing the same open arm. The open arms measured 112 cm × 10.4 cm, and the closed arms were 113 cm × 10.1 cm × 36.5 cm. Each animal was tested for a total of five minutes. After each animal was tested, the maze was cleaned with 70% ethanol. Observed behaviors were the total number of two and four paw entries into the open and closed arms, as well as the total time spent in each arm or the central platform.

#### 2.6.2 Porsolt swim task

A task designed to measure depressive‐like behavior and learned helplessness, animals were placed in buckets of water, at 25°C, and were observed for a total of 5 min each day (Can et al., 2011). Water depth was maintained at 17 inches in 22 × 18‐inch plastic cylinder. Animals were placed in the center of the bucket and were dried off with paper towel and placed back into their home cages for approximately 24 hr prior to the second day of PST testing. Observed behavior included latency‐to‐float and the total time spent immobile. Immobility was defined as swimming cessation—any movement involved in keeping the animal's head above water or to push itself from bumping into the wall were excluded.

### 2.7 Brain dissections

At nineteen weeks, animals were euthanized via intraperitoneal injection of 75 mg of ketamine (100 mg/ml, Ketalean, CDMV Inc.) and 5 mg xylazine (100 mg/ml, Sigma, USA) per kg of body weight. Brains were harvested immediately and frozen on dry ice and were subsequently stored at −80°C until genetic analysis. Brain regions were determined utilizing Paxinos and Watson's Rat Brain Atlas (Paxinos et al., 1997). The prefrontal cortex was delineated by a +2.2 mm anterior–posterior (AP) position from bregma. All dissections were conducted in sterile petri dishes on top of ice.

### 2.8 Primer design

Primers were designed using Primer3 and BLAST; sequences and accession numbers are listed in Supplementary Information, Table S1. Primer validation was conducted utilizing serial dilutions of cDNA, and specificity was analyzed using melt curves post amplification (Livak & Schmittgen, 2001). Genes were chosen based on a whole genome microarray analysis that were relevant to the metabotropic glutamate receptor pathway; β 1, 2, and 3 adrenergic receptor signaling; serotonin receptor signaling; methylation; neural differentiation and growth; and glucocorticoid receptors (Mychasiuk et al., 2011).

**TABLE 1:** Sex main effects on EPM and PST

Sex	Open Arms	Central	Open Arms−2 Paws	Open Arms−4 Paws	Immobility Day 2
Males	21.4 ± 7.45	104.54 ± 11.28	7.13 ± 0.89	1.93 ± 2.43	174.68 ± 7.10
Females	*53.89 ± 10.25*	*73.47 ± 8.90*	*3.80 ± 0.64*	*4.13 ± 2.47*	*212.96 ± 6.78*

Note

The mean and S.E.M. of measured behavior with sex main effects on EPM and PST measures. Open arms and closed arms are measured in seconds, 2‐ and 4‐paw entries are measured in total number of entries, and immobility day 2 is measured in seconds. Males are presented on top, and females are presented in italics.

### 2.9 RNA extraction and complimentary DNA synthesis

Each PFC was weighed and then mechanically homogenized for two 2‐min cycles at 30 Hz in a TissueLyser (Qiagen) with TRI Reagent (Sigma‐Aldrich; 1 ml/50 mg of tissue) as previously described (Grandbois et al., 2016; Khurana et al., 2019; Nguyen et al., 2015). Supernatant was added to 200 µl of chloroform, vortexed, and incubated at room temperature (RT) for 15 min. After centrifugation, the top aqueous phase was precipitated with 500 µl of isopropanol and pellets were washed with of 70% ethanol. After a final centrifugation, RNA pellets air dried and then resuspended in 20 µl of diethylpyrocarbonate (DEPC)‐treated nuclease‐free water and were then placed on a ThermoMixer R (Eppendorf) for 10 min at 1,000 rpm and 37°C. Concentration of total RNA was measured using the spectrophotometric measurement of the absorbance at 260 nm (Nanodrop ND‐1000, Nanodrop Technologies). RNA suspensions were stored at −80°C for long‐term storage.

Two µg of total RNA was treated with DNAse I (Sigma‐Aldrich) to remove genomic DNA. Complimentary DNA (cDNA) was synthesized by adding random primers (Roche Diagnostics) and Mu‐MLV reverse transcriptase (Promega) as per manufacturer guidelines. A negative control was prepared with no reverse transcriptase. Final concentration of cDNA samples was 0.04 µg/µl.

### 2.10 RT‐qPCR

Alteration in gene expression between naïve and DEX was analyzed by comparing expression of target genes with multiple housekeeping genes using 2^ΔΔCQ. CQ values were measured utilizing the QuantStudio5 Real‐Time PCR System (Applied Biosystems) to compare samples from the naïve and DEX animals (*n* = 5–8). 15 µl reaction volumes were used with cDNA (0.4 ng/µl), DEPC water, forward and reverse primers (1.2 µM/µl), and SYBR green master‐mix (SensiFAST SYBR Lo‐ROX, Bioline, Froggabio).

### 2.11 Statistical analysis

All statistical analyses for behavioral and genetic comparisons were carried out using IBM SPSS v20.0. Datasets were tested for normality and homogeneity of variance using Shapiro–Wilk and Levene's test, *p* > .05. General linear model analysis of variance (2 × 2 ANOVAs) were conducted, otherwise Welch's test was utilized with alpha levels set to *p* = .05. All results are presented in mean ± standard error of the mean (S.E.M). Post hoc analyses were conducted where appropriate, using Tukey's Honestly Significant Difference (HSD). Statistical analysis examining naïve and sham controls is located in the supplemental information (Table  S2).

**TABLE 2:** Gene Expression in the PFC

Relative Gene Expression in the Prefrontal Cortices of DEX Offspring		
GENE	SEX	FOLD CHANGE 2^ΔΔCT + *SEM*
**Glucocorticoid Receptors**		
NR3C1	Males Females	1.17 ± 0.16 1.02 ± 0.15
NR3C2	Males Females	0.97 ± 0.21 1.07 ± 0.34
**Methylation**		
COMT	Males Females	**1.28 ± 0.11** 0.84 ± 0.17
DNMT3b	Males Females	***1.80 ± 0.32*** ***1.12 ± 0.08***
**Glutamate Signaling**		
GRM4	Males Females	1.64 ± 0.43 1.53 ± 0.69
SLC1A2	Males Females	1.00 ± 0.06 0.95 ± 0.14
GRIA2	Males Females	1.29 ± 0.19 1.15 ± 0.19
GRM2	Males Females	1.57 ± 0.41 1.00 ± 0.22
**Calcium Signaling**		
RYR2	Males Females	***1.57 ± 0.25*** ***0.81 ± 0.09***
CACNB2	Males Females	1.12 ± 0.11 0.89 ± 0.16
CACNA1B	Males Females	1.23 ± 0.14 0.95 ± 0.16
PLCH2	Males Females	1.55 ± 0.38 1.08 ± 0.12
RYR1	Males Females	1.34 ± 0.31 1.20 ± 0.14
**Neural Transmission**		
SNAP25	Males Females	**1.62 ± 0.20** 1.14 ± 0.26
**Neuronal Growth & Differentiation**		
MYT1L	Males Females	1.00 ± 0.13 1.18 ± 0.28
LSAMP	Males Females	**1.45 ± 0.20** 1.06 ± 0.10
**Lysosomal Homeostasis**		
MBTPS1	Males Females	***1.40 ± 0.13*** ***0.86 ± 0.09***

Note

Fold change of DEX males and females compared with naïve controls. Bolded numbers, highlight in red, are significant compared with controls. Sex by treatment interactions are in italics and are shaded blue. All significances are *p* < .05.

## 3 RESULTS

### 3.1 Behavioral tasks

**FIGURE 2:**
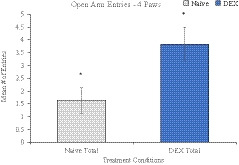
Treatment main effect of the average number of 4‐paw entries into the open arm of the EPM. DEX offspring (3.84 ± 0.60) were more active than naïve controls (1.64 ± 0.49), *Denotes *p* < .05

Observational data for the EPM measured exploratory activity as an indicator for general anxiety. A main effect of sex was found on several measures: total time spent in the open arms, *F* (1, 28) = 6.576, *p* = .016, where females spent more time (*M* = 53.89 ± 10.25) than males (*M* = 21.4 ± 7.45); total time spent on the central platform, *F* (1, 28) = 4.675, *p* = .039, with males spending more time (*M* = 104.54 ± 11.28) than females (*M* = 73.47 ± 8.90); total number of 2‐pawed entries into the open arms, *F* (1, 28) = 9.254, *p* = .005, with males more active (*M* = 7.13 ± 0.89) than females (*M* = 3.80 ± 0.64); and the total number of 4‐pawed entries into the open arms, *F* (1, 28) = 6.026, *p* = .021, with females more active (*M* = 4.13 ± 2.47) than males (*M* = 1.93 ± 2.43; Table 1). A main effect of treatment showed fewer 2‐pawed entries from the DEX offspring compared with controls (1.00 ± 0.31) into the closed arms compared with naïve offspring (2.00 ± 0.36), *F* (1, 28) = 4.241, *p* = .049. A main effect of treatment also showed the DEX offspring entered the open arms with 4‐paws more often than the naïve controls, Welch's *F* (1, 27.995) = 7.328, *p* = .011 (Figure 2). Within the open arms with 4‐paws measure, there was also a main effect of sex *F* (1, 38) = 6.062, *p* = .018.

**FIGURE 3:**
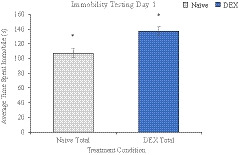
Treatment main effect of the average time (s) spent immobile on testing day 1 in the PST. DEX offspring (137.25 ± 5.94) were significantly more immobile than their naïve counterparts (107.38 ± 6.32), * denotes *p* < .05

In the PST, animals’ immobility and latency‐to‐float were measured in relation to despondency. There was a main effect of sex on immobility in day 2, *F* (1, 28) = 15.198, *p* = .001 where females were more inactive (*M* = 212.96 ± 6.78) than males (*M* = 174.68 ± 7.10; Table 1). A main effect of treatment was also shown, where DEX offspring were significantly more immobile than naïve offspring on day 1 of testing, *F* (1, 28) = 10.572, *p* = .003 (Figure 3), whereas sex effects were trending, but not significant at *p* < .05.

### 3.2 Genetic analysis

**FIGURE 4:**
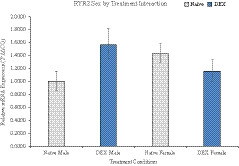
Interaction of sex by treatment in gene expression of Ryanodine Receptor 2. DEX male offspring have an increase in gene expression compared with controls, whereas DEX females show a decrease in overall expression

**FIGURE 5:**
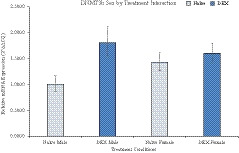
Interaction of sex by treatment in gene expression of DNA‐Methyltransferase 3‐beta. The gene expression of DEX male offspring is significantly increased in comparison with naïve males. DEX females do not show a significant change in expression; however, female animals overall show higher expression to naïve males

**FIGURE 6:**
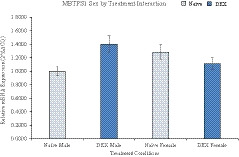
Interaction of sex by treatment in gene expression of membrane‐bound transcription factor peptidase site 1. Naïve females show baseline levels of higher gene expression; however, DEX females show lower levels of gene expression and DEX males show an increase

Seventeen genes were analyzed and several genes were dysregulated in the PFC associated with neurotransmission and methylation (Table 2). Synaptosome Associated Protein 25 (SNAP25) was increased in DEX males compared with naïve controls with a fold change of 1.62 ± 0.20 (2^ΔΔCQ ± S.E.M.). Ryanodine Receptor 2 (RYR2) had a sex by treatment interaction, *F* (1, 28) = 8.611, *p* = .009, η^2^ = 0.324 (Figure 4). Limbic system‐associated membrane protein (LSAMP) had increased expression in DEX males (1.45 ± 0.20), as did catechol‐O‐methyltransferase (COMT; 1.28 ± 0.11). DNA‐methyltransferase 3 Beta (DNMT3b) displayed a sex by treatment interaction, *F* (1, 17) = 5.555, *p* = .031, η^2^ = 0.246 (Figure 5). As well, membrane‐bound transcription factor peptidase site 1 (MBTPS1) also displayed an interaction, *F* (1, 18) = 11.895, *p* = .003, η^2^ = 0.398 (Figure 6).

## 4 DISCUSSION

The prefrontal cortex is linked to inhibition and decision‐making, often referred to as cognitive control, when faced with novel and stressful situations (Miller & Cohen, 2001; Ridderinkhof, Van Den Wildenberg, Segalowitz, & Carter, 2004). It permits for a change in behavioral response upon evaluation of the situation and applying context (Levy & Wagner, 2011). Provided normal neural development, the PFC is key to making appropriate decisions and responses.

Here, we evaluate the potential for chronic late gestational prenatal stress to influence PFC function in offspring. Our results lead us to the conclusion that stress can program behavioral changes, considering the increase in immobility of DEX animals during the PST. Historically, the PST has been an indicator of learned helplessness and despondency (Kraeuter, Guest, & Sarnyai, 2019). Recently, however, the research community has shifted its focus of the PST as a depressive measure, but to that of adaptive coping strategies when faced with a stressful situation (Molendijk & de Kloet, 2019). DEX offspring also provided indication of increased exploratory activity, with significant increases in the number of 4‐paw entries into the open arms of the EPM, which goes against the animals’ natural tendency toward thigmotaxis (Treit & Fundytus, 1988). In consideration of the adaptive theory of fetal programming, these behaviors indicate fetal programming of the PFC may lead to changes in typical response to novel situations that may increase survival, or influence other brain regions, such as the hippocampus and amygdala. Arguably, these behaviors are not always to the benefit of the offspring, such as in the thrifty phenotype, but are situationally dependent. Exploratory behavior may indicate the animal is not anxious and, however, poses an increased risk of predation to the animal. Conservation of energy as a coping strategy may lead to loss of life through hypothermia or a missed opportunity for escape. Similar to the results of the current study, Sprague Dawley rats exposed to late gestation GC displayed passive coping strategies on the PST at the age of 9 weeks (Xu et al., 2018). Another study with Sprague Dawley rats produced a sex‐specific effect on the PST, with females spending significantly more time immobile than other treatment conditions (Hiroi et al., 2016). In one study utilizing the same dosage and timing of DEX treatment as the current study, the authors found no significant behavior during the EPM trial; however, their animals were aged 10 weeks, which could implicate behavioral differences may be age‐dependent in manifestation (Zeng et al., 2015). HPA axis function and behavioral responses in GC‐exposed adult offspring tend to vary in literature depending upon sex, age, and dosage, but also the testing paradigms (Cartier et al., 2016; McGowan & Matthews, 2018).

In combination with the behavioral changes, overexpression of multiple genes was reported. Changes in methylation and genetic expression within the PFC and other brain regions have been linked to adverse behavior that include depression, anxiety, decreased locomotor activity, and cognition (Constantinof et al., 2019; Turner et al., 2010; Weaver et al., 2004). In the current study, COMT was overexpressed in DEX males, a gene that encodes for methyltransferase that commonly inactivates neurotransmitters such as dopamine and noradrenaline. COMT adds methyl groups to the neurotransmitters, preventing their ability to bind to their respective receptors, thereby limiting neural response to emotional stimuli, such as fear and anxiety. In another methyltransferase gene, the DNMT3b interaction shows baseline sex variation and a significant increase in expression of DEX male offspring. This gene regulates neuronal processes, development, and plasticity (Bayraktar & Kreutz, 2018). SNAP25 was also overexpressed in DEX males, a gene involved in neurotransmitter release from vesicles into the synapse; combined with increased expression of COMT, the neurotransmitter signaling pathways in the PFC of DEX animals, particularly males, is overactive. LSAMP, a third neuronally relevant gene, was overexpressed in DEX males and is involved in neuronal growth and tumor suppression. Complete deletion of LSAMP in genetic models leads to significant disruption of anxiety‐related behavior in the EPM, similar to hyperactivity (Catania et al., 2008). Also involved in neural transmission and synaptic plasticity is calcium receptor RYR2. RYR2 is link to memory processing through calcium release, whereby an increase in calcium concentrations can activate long‐term potentiation and solidify memories (Del Prete et al., 2014). The RYR2 interaction seen in our results indicates a reversal in sex‐specific gene expression. Naïve females have increased relative expression than naïve males, whereas DEX males are overexpressed and DEX females drop in expression to levels closer to the naïve males. The last dysregulated gene was MBTPS1, which followed same pattern as RYR2, where the sex difference in naïve animals switches its pattern in the DEX offspring with DEX males overexpressing and DEX females lowering expression. MBTPS1 cleaves pro‐BDNF (brain‐derived neurotrophic factor) into a truncated form (Peng et al., 2018).

## 5 CONCLUSIONS

In summary, the results from the current study show that the prefrontal cortex is a relevant area of interest when exploring prenatal stress and fetal programming. Chronic glucocorticoid exposure during late gestation influences the genetic profile of this cognitively relevant region and influences adaptive behavioral phenotypes in novel situations in the adult offspring.

## CONFLICT OF INTEREST

The authors declare there are no conflicts of interest.

## AUTHOR CONTRIBUTION

CL and JG contributed substantially to conducting the experiments, collecting the behavioral observations, analysis, and interpretation. CL, JG, AM, and SK contributed to data collection and analysis. CL, SK, ST, and TCT contributed to data interpretation and writing/editing of manuscript. TCT contributed substantially to the study design and project oversight.

## Supporting information

Table S1‐S2

## Data Availability

The data that support the findings of this study are available upon reasonable request.
