# Supplementary material for: Late gestational exposure to dexamethasone and fetal programming of abnormal behavior in Wistar Kyoto rats
Source: Brain Behav. 2021 Feb 2;11(4):e02049. doi: 10.1002/brb3.2049 (PMC8035474; doi:10.1002/brb3.2049)
Supplement: Supplementary file 1 — Table S1‐S2 [file BRB3-11-e02049-s001.docx]

**Supplementary Information**

Table 1. Primer Design

| Pathway | Gene | Accession # | Forward Primer (5’ → 3’) | Reverse Primer (5’ → 3’) |
| --- | --- | --- | --- | --- |
| Methylation | COMT | NM_012531.2 | TTCAAGCGTCGGGATCGG | TGCAGCACGTACTCAAACCA |
|  | DNMT3b | NM_001003959.1 | GATGAGGAGAGCCGAGAACG | CAGAGCCCACCCTCAAAGAG |
| Glutamate signaling | GRM4 | NM_022666.1 | CAGTGCGAGCAGCTAAGGG | GAGAATGGCTCCGGTCACTC |
|  | SLC1A2 | NM_001035233.1 | GTCAATGCCGCACACAACTC | GAATGGATGCAGGGGATGGT |
|  | GRIA2 | NM_001083811.1 | GCATCGCCACACCTAAAGGA | TCCTTGGAATCACCTCCCCC |
|  | GRM2 | NM_001105711.1 | TTCCCTCCTCAGTCTTCCACT | CCTCCATAGGAAGCAGCGTT |
| Calcium signaling | RYR2 | NM_001191043.1 | CACTAAGCAGCATCCTGTGC | CTTCGGTCTTGGCTTCTCAGT |
|  | CACNB2 | NM_053851.1 | CAGCTGCACTGTCGGAATCT | AGTCATTCCATTTTTGCCCTGA |
|  | CACNA1B | NM_001195199.1 | GGGCTAATCTGCCCCAGAAG | GAGAGCCGCATAGACCTTCC |
|  | PLCH2 | XM_017593885.1 | AAATCCCAGAGTGCCCATCC | ATCGTTCCACCACAGGCAAA |
|  | RYR1 | XM_017590442.1 | GGGCCATAACAACGGTGAGA | TCAGACGACATCGCAGTCAC |
| Glucocorticoid receptors | NR3C1 | NM_012576.2 | TGCTGGAGGTGATTGAACCC | TCACTTGACGCCCACCTAAC |
|  | NR3C2 | NM_013131.1 | CAGTGCACAGTCCCATCACT | GGACTTGAAAGAGGGGAGCC |
| Neural transmission | SNAP25 | NM_001270575.1 | ATGTTGGATGAGCAAGGCGA | TCGGCCTCCTTCATGTCTTG |
| Neuronal differentiation, growth | MYT1L | NM_053888.1 | TGTGGAGCCAGCCATACAAG | TGGGGCTGTTTATCTTGCGT |
|  | LSAMP | NM_017242.1 | CACTGAGGAACACTACGGCA | ACCCGGGTCTGAAAAGGACT |
| Lysosomal homeostasis | MBTPS1 | NM_053569.1 | GCGAGTAAACATCCCCCGAA | CCCAAATCTAGCAGGAGCCC |
| Reference genes | Aanat | XM_006247792.2 | GACAAGACGTCTCCCTCTGG | GGTGGATGCTCAACATGGGT |
|  | CycA | NM_017101.1 | CAGACGCCGCTGTCTCTTTTC | CGTGATGTCGAAGAACACGGT |
|  | Ywhaz | NM_013011.3 | GGCAGAGCGATACGATGACA | AAGATGACCTACGGGCTCCT |

All primers listed were designed using Primer3 and BLAST. Annealing temperature was 58°C.

Table 2. Naïve versus Sham Control Analysis

| One-Way ANOVA (Welch's) | | | | | | | | | |
| --- | --- | --- | --- | --- | --- | --- | --- | --- | --- |
|  |  |  |  |  |  |  |  |  |  |
|  | | **F** | | **df1** | | **df2** | | **p** | |
| Latency Day 1 |  | 2.1851 |  | 1 |  | 18.95 |  | 0.156 |  |
| Immobility Day 1 |  | 1.0386 |  | 1 |  | 8.00 |  | 0.338 |  |
| Latency Day 2 |  | 1.4139 |  | 1 |  | 13.08 |  | 0.256 |  |
| Closed Arms |  | 0.5539 |  | 1 |  | 18.33 |  | 0.466 |  |
| Open Arms |  | 1.3239 |  | 1 |  | 12.71 |  | 0.271 |  |
| Central Platform |  | 0.0232 |  | 1 |  | 17.87 |  | 0.881 |  |
| Open Arms 2 Paws |  | 0.7037 |  | 1 |  | 19.00 |  | 0.412 |  |
| Open Arms 4 Arms |  | 0.1392 |  | 1 |  | 18.58 |  | 0.713 |  |
| Open Arms Total |  | 0.5140 |  | 1 |  | 18.88 |  | 0.482 |  |
|  | | | | | | | | | |

Analysis of variance was conducted using jamovi 1.6.6.
